# Cost-effectiveness analysis of pembrolizumab plus trastuzumab and chemotherapy as first-line treatment for HER2-positive gastric or gastroesophageal junction adenocarcinoma

**DOI:** 10.3389/fmed.2026.1905243

**Published:** 2026-09-15

**Authors:** Haoyu Yang, Weihao Ge, Shuxian Jiang, Xin Lan, Xinting Du

**Affiliations:** School of Health Economics and Management, Nanjing University of Chinese Medicine, Nanjing, Jiangsu, China

**Keywords:** cost-effectiveness, gastric/gastroesophageal junction adenocarcinoma, HER2-positive, pembrolizumab, trastuzumab

## Abstract

**Objectives:**

The final analysis of KEYNOTE-811 demonstrated that pembrolizumab plus trastuzumab and chemotherapy significantly improved both overall survival (OS) and progression-free survival (PFS) compared to trastuzumab plus chemotherapy alone. Based on these latest findings, this study evaluated the cost-effectiveness of pembrolizumab plus trastuzumab and chemotherapy from the perspective of the Chinese healthcare system.​

**Design:**

Using a partitioned survival model, this study compared quality-adjusted life years (QALYs), total costs, and incremental cost-effectiveness ratios (ICERs) between pembrolizumab plus trastuzumab and chemotherapy versus trastuzumab plus chemotherapy alone. Efficacy and safety data were derived from KEYNOTE-811, local databases, and relevant literature. To validate the robustness of the model results, sensitivity analyses were conducted. Additionally, subgroup analyses were conducted specifically for the patient population with PD-L1 expression ≥1%.​

**Interventions:**

Pembrolizumab plus trastuzumab and chemotherapy.

**Outcome measures:**

Total costs, life years, quality adjusted life years (QALYs), incremental QALYs and incremental cost-effectiveness ratio (ICER).

**Results:**

In the intention-to-treat (ITT) population, pembrolizumab plus trastuzumab and chemotherapy demonstrated a gain of 0.56 QALYs compared to trastuzumab plus chemotherapy alone, with an additional cost of $322,653.47, resulting in an ICER of $571,997.42/QALY. In the PD-L1 ≥ 1 subgroup, the pembrolizumab combination arm showed a QALY improvement of 0.38, yielding an ICER of $1,178,714.74 per QALY.​

**Conclusion:**

From the perspective of Chinese healthcare system, the addition of pembrolizumab to trastuzumab-chemotherapy as first-line therapy failed to demonstrate cost-effectiveness for HER2-positive gastric/gastroesophageal junction adenocarcinoma across both the ITT population and PD-L1 ≥ 1 subgroup.

## Strengths and limitations of this study

Adopted a partitioned survival model with three mutually exclusive health states (PFS, PD, death), 3-week cycles and lifetime horizon, ensuring the model fits the natural course of HER2-positive gastric/gastroesophageal junction adenocarcinoma.

Derived clinical efficacy and safety data from the global, multicenter KEYNOTE-811 phase 3 trial, with large sample size and high evidence level to support model reliability.

Extrapolated long-term survival data using parametric distributions, introducing uncertainty from model assumptions beyond trial follow-up.

Only included grade ≥3 adverse events for cost and disutility calculation, possibly underestimating total medical costs.

## Introduction

Gastric cancer (GC), including gastroesophageal junction cancer (GEJC) represent a global health challenge, being the fifth most prevalent cancer type worldwide with attributable fractions of 5.6% for incidence and 7.7% for mortality (1). The Chinese population experienced 358,700 incident cases in 2022, accompanied by >260,400 deaths, underscoring its substantial public health impact. Notably, >95% of GC cases are adenocarcinomas (2), which typically progress silently - early clinical manifestations are limited to nonspecific upper gastrointestinal symptoms, leading to delayed diagnosis in 80%–90% of cases until advanced disease stages and consequent poor prognosis (5-year survival: 6%) (3).

The National Comprehensive Cancer Network (NCCN) guidelines endorse platinum-fluorouracil chemotherapy regimens including folinic acid/5-fluorouracil/oxaliplatin (FOLFOX) and capecitabine/oxaliplatin (CAPOX) as first-line therapy for advanced GC/GEJC (4), though its clinical benefits remain modest with considerable toxicity. The advent of programmed death-1/programmed death-ligand 1 (PD-1/PD-L1) inhibitors has revolutionized cancer treatment paradigms (5–7). While nivolumab (CheckMate-649) and pembrolizumab (KEYNOTE-859) have shown efficacy in HER2-negative disease, patients with HER2-positive GC/GEJC adenocarcinoma continue to represent an underserved population with limited treatment options.

Pembrolizumab, an anti-PD-1 monoclonal antibody, blocks the interaction between PD-1 and PD-L1, thereby restoring T-cell-mediated antitumor activity and enhancing immune system-mediated tumor killing. The KEYNOTE-059 and KEYNOTE-062 trials provided pivotal evidence supporting pembrolizumab's approval in GC/GEJC (8). Trastuzumab is approved for HER2-positive GC/GEJC patients (9). Both pembrolizumab and trastuzumab have demonstrated favorable efficacy in their respective clinical studies for GC/GEJC. However, limited evidence exists regarding the progression-free survival (PFS) and overall survival (OS) benefits of dual PD-1 and HER2 blockade combined with chemotherapy for HER2-positive GC/GEJC adenocarcinoma.

To address this, the global KEYNOTE-811 trial (conducted across 168 medical centers in 20 countries) evaluated pembrolizumab plus trastuzumab and chemotherapy as first-line treatment for HER2-positive GC/GEJC adenocarcinoma. Results demonstrated significant improvements versus placebo plus trastuzumab-chemotherapy in both PFS (ITT population: 10.0 vs. 8.1 months; PD-L1 CPS ≥1 population: 10.9 vs. 7.3 months) and OS (ITT population: 20.0 vs. 16.8 months; PD-L1 CPS ≥1 population: 20.1 vs. 15.7 months) (10, 11). The incidence of grade ≥3 adverse events was comparable between pembrolizumab (58%) and placebo (51%) groups. Therefore, pembrolizumab combined with trastuzumab and chemotherapy appears to be an attractive first-line treatment option for HER2-positive GC/GEJC adenocarcinoma patients.​

Although the KEYNOTE-811 trial has demonstrated improved clinical outcomes with pembrolizumab combination therapy in HER2-positive GC/GEJC adenocarcinoma, the high cost of pembrolizumab may limit its accessibility, potentially offsetting its antitumor efficacy. Currently, pembrolizumab gained approval in China for HER2-positive GC/GEJC adenocarcinoma in June 2024. Therefore, pharmacoeconomic evaluation is warranted to assess its cost-value ratio and provide evidence-based references for healthcare decision-makers.

## Methods

This health economic evaluation was conducted in full compliance with the Consolidated Health Economic Evaluation Reporting Standards (CHEERS) checklist (12).

### Patients and interventions

The hypothetical target population of this study consisted of patients aged ≥18 years with pathologically confirmed unresectable or metastatic GC or GEJC adenocarcinoma, previously untreated, HER2-positive status, measurable lesions assessed by RECIST v1.1, ECOG PS 0 or 1, life expectancy >6 months, adequate organ function, and sufficient tumor samples for PD-L1 and microsatellite instability assessment, consistent with the patient characteristics of the KEYNOTE-811 trial. On day 1, patients received pembrolizumab 200 mg or placebo intravenously + trastuzumab (initial 8 mg/kg loading dose, followed by 6 mg/kg every 3 weeks) + chemotherapy [cisplatin plus 5-fluorouracil (fp) or capecitabine plus oxaliplatin (CAPOX)], administered in 3-week cycles for up to 35 cycles, until disease progression, unacceptable toxicity, withdrawal of informed consent, or study discontinuation. According to data disclosed in KEYNOTE-811, the proportions of patients in the pembrolizumab + trastuzumab and chemotherapy group who received cisplatin and 5-fluorouracil (5-FU) or capecitabine and oxaliplatin were 15% and 85%, respectively. In the trastuzumab and chemotherapy group, these proportions were 14% and 86%, respectively. Unit costs of all chemotherapy agents were extracted from the Yaozh database, and total per-cycle chemotherapy costs were calculated according to the above weighted distribution.

All patients received second-line treatment after disease progression. Based on the NCCN Clinical Practice Guidelines in Oncology version (13) and systemic treatment information provided by the KEYNOTE-811 trial, we assumed the second-line regimen to be chemotherapy-based treatment (paclitaxel, docetaxel, or irinotecan monotherapy). Palliative supportive care was provided for patients with disease progression in advanced stages. Baseline characteristics between the Pembrolizumab and Chemotherapy groups were shown in Supplementary Table S1.

### Model construction

This study developed a partitioned survival model with three mutually exclusive health states to evaluate the initial treatment decision of pembrolizumab or placebo combined with trastuzumab plus chemotherapy for HER2-positive GC/GEJC adenocarcinoma. The three mutually exclusive health states were: progression-free survival (PFS), progressed disease (PD), and death. As illustrated in Figure 1, all patients entered the model in the PFS state. During each 3-week model cycle, patients either remained in their current state or transitioned to the next health state. The model operated with 3-week cycles over a lifetime horizon, capturing >99% of patients’ complete life expectancy. The key outputs of the model included total costs, quality-adjusted life years (QALYs), and incremental cost-effectiveness ratios (ICERs). The model was constructed and data analysis was performed using Excel 2019.

**Figure 1:**
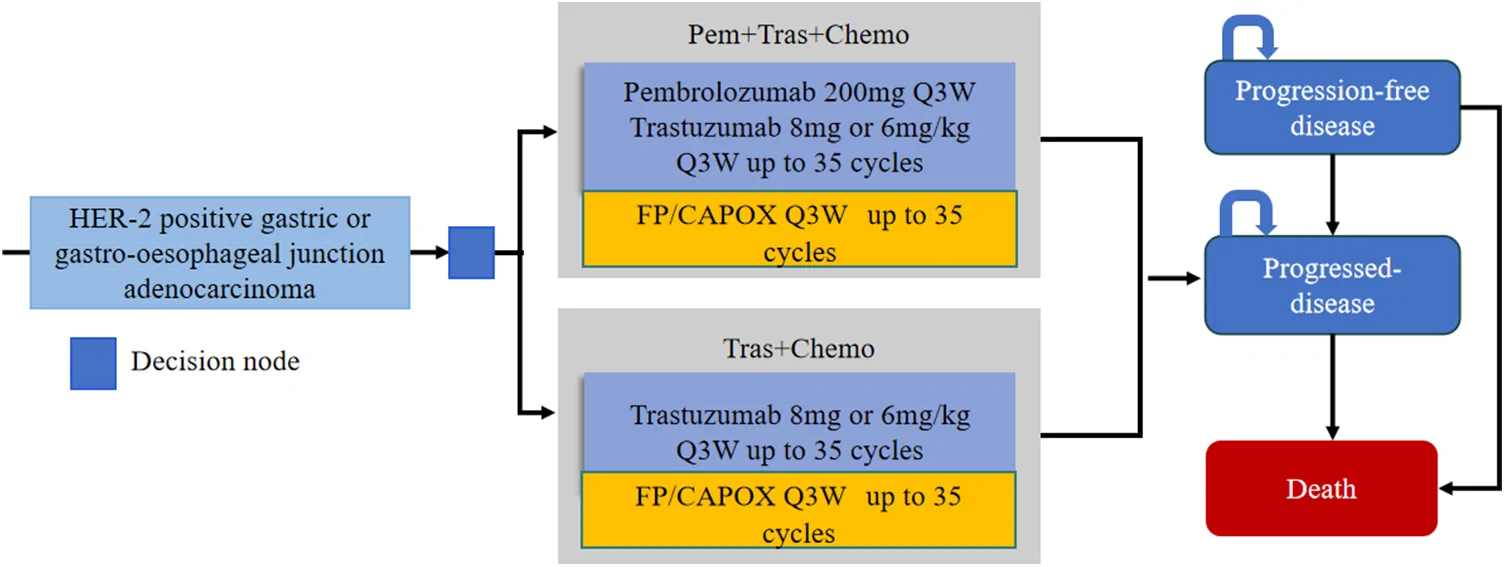
Diagram of partitioned survival model. Pem, pembrolizumab; Tras, trastuzumab; Chemo, chemotherapy; FP, cisplatin and 5-fluorouracil; CAPOX, capecitabine and oxaliplatin.

### Clinical effectiveness

The survival data for pembrolizumab and placebo groups were derived from the KEYNOTE-811 trial and extrapolated using the statistical method described by Guyot et al. (14) The GetData Graph Digitizer software was employed to extract data points from the original Kaplan–Meier (K-M) survival curves. These extracted data points were then used to fit and extrapolate corresponding survival functions for long-term clinical outcomes, utilizing the following parametric distributions: Exponential, Gamma, Gompertz, Weibull, Log-logistic, and Log-normal (15). The optimal fitting distribution was selected based on the Akaike Information Criterion (AIC) and Bayesian Information Criterion (BIC), Supplementary Figures S1,S2 illustrates the OS and PFS fitting curves for both the pembrolizumab + trastuzumab + chemotherapy and trastuzumab + chemotherapy groups, whereas Supplementary Table S2 provides a comprehensive description of the survival model selection, and Supplementary Table S3 presents the survival parameters used in the model.

### Medical costs

This study only included direct medical costs in the model analysis, including drug costs, follow-up management costs (including bed charges, nursing fees, diagnostic fees, and intravenous infusion fees), adverse reaction management costs, and palliative care costs. Pembrolizumab was priced as a fixed 200 mg dose per cycle, whereas trastuzumab and chemotherapy doses were calculated using standard anthropometric parameters (body weight: 65 kg, body surface area: 1.72 m^2^). The costs of pembrolizumab, trastuzumab, and chemotherapy drugs were obtained from Yaozh.com (https://www.yaozh.com/), while follow-up management costs, adverse reaction management costs, and palliative care costs were derived from relevant literature data (16, 21). To simplify the model, this study only considered grade ≥3 treatment-related severe adverse reactions, with calculations limited to the first cycle.

The utility values for PFS and PD states were obtained from published studies, with values of 0.797 and 0.577, respectively (19). Additionally, this study incorporated the disutility associated with adverse reactions (22, 23), assuming it occurred in the first cycle. Both costs and utilities were discounted at a rate of 5%. The willingness-to-pay (WTP) threshold was set at three times China's 2024 per capita gross domestic product (GDP) ($40,334.05/QALY). This 3× GDP standard is WHO-recommended for middle-income countries and widely adopted in domestic oncology pharmacoeconomic studies for policy evaluation of anti-tumor biologics. The parameters considered in the model are presented in Table 1.

**Table 1:** Key model parameters.

Parameters	Base-case value	Ranges	Distribution	Source
**Cost ($/cycle)**				
Pembrolizumab	5,229.24	4,183.39–5,229.24	Gamma	yaozh database
Trastuzumab-1	579.28	463.42–579.28	Gamma	yaozh database
Trastuzumab-2	434.46	347.57–434.46	Gamma	yaozh database
Fluorouracil	6.88	5.50–6.88	Gamma	yaozh database
Cisplatin	0.69	0.55–0.69	Gamma	yaozh database
Capecitabine	91,985.6	73,588.48–91,985.60	Gamma	yaozh database
oxaliplatin	107.33	85.86–107.33	Gamma	yaozh database
Nap_paclitaxel	473.85	426.47–521.24	Gamma	yaozh database
Ramucirumab	4.257	3.05–5.11	Gamma	yaozh database
Supportive care	359	269.25–448.75	Gamma	(20)
**Routine follow-up per unit**				
Enhanced CT	171.03	145.82–218.74	Gamma	(23)
Bed	6.6	5.22–7.83	Gamma	(21)
Diagnosis	3.14	1.55–4.66	Gamma	(21)
Intravenous injection	1.73	1.55–2.14	Gamma	(21)
Care	3.77	2.98–4.47	Gamma	(21)
**Management of adverse event**				
Diarrhoea	3.51	2.81–4.21	Gamma	(20)
Anemia	336.63	269.30–403.96	Gamma	(20)
Neutrophil count decreased	454.26	363.41–545.11	Gamma	(20)
Platelet count decreased	1,054.22	843.38–1,265.06	Gamma	(20)
Neutropenia	454.26	363.41–545.11	Gamma	(20)
**Utility values/Disutility values**				
PFS	0.797	0.638–0.956	Beta	(18)
PD	0.577	0.462–0.692	Beta	(18)
disuDiarrhoea	0.32	0.26–0.38	Beta	(23)
disuAnanemia	0.07	0.06–0.08	Beta	(18)
disuNeutrophil count decreased	0.20	0.16–0.24	Beta	(22)
disuPlatelet count decreased	0.25	0.23–0.28	Beta	(23)
disuNeutropenia	0.20	0.16–0.24	Beta	(17)
**Others**				
Discount rate	5%	0%–8%	Beta	(29)

### Sensitivity analysis

To evaluate the robustness of the model outcomes, we conducted both one-way sensitivity analysis and probabilistic sensitivity analysis. The one-way sensitivity analysis calculated ICER values using the upper and lower bounds of pre-specified distributions to examine the impact of various parameters within defined ranges (when ranges were unknown, mean ±20% was used as the boundaries) on ICER values, with results presented as tornado diagrams. In the probabilistic sensitivity analysis, 1,000 Monte Carlo simulations were performed to simultaneously adjust all parameters’ effects on ICER, which helped assess the probability of the treatment regimen being cost-effective and the QALYs at different willingness-to-pay thresholds. In the model, cost parameters followed Gamma distributions, while utility values, adverse event rates, and discount rates followed Beta distributions. The results were presented as cost-effectiveness acceptability curves and scatter plots.

## Results

### Base-Case analysis

The base case analysis results are presented in Table 2. In the ITT population, compared with placebo plus trastuzumab and chemotherapy, pembrolizumab plus trastuzumab and chemotherapy provided an additional 0.56 QALYs at an incremental cost of $322,653.47, resulting in an ICER of $571,997.42 per QALY. In the PD-L1 ≥ 1 population, pembrolizumab plus trastuzumab and chemotherapy provided an additional 0.38 QALYs compared with placebo plus trastuzumab and chemotherapy, with an incremental cost of $453,095.44, yielding an ICER of $1,178,714.74 per QALY.

**Table 2:** Base-case analysis results.

Group	Cost/$*	QALYs*	Incremental cost/$	Incremental QALYs	ICER/($/QALY)
**ITT Population**					
Pembrolizumab + trastuzumab + chemotherapy group	1,792,323.91	2.11	322,653.47	0.56	571,997.42
trastuzumab + chemotherapy group	1,469,670.44	1.54	NA	NA	NA
**PD-L1** **≥** **1 Population**					
Pembrolizumab + trastuzumab + chemotherapy group	1,820,529.58	2.24	453,095.44	0.38	1,178,714.74
trastuzumab + chemotherapy group	1,367,434.14	1.86	NA	NA	NA

ICER, incremental cost-effectiveness ratio; QALY, quality-adjusted life-year.

* *P*-value < 0.0001.

### Sensitivity analysis

The one-way sensitivity analysis demonstrated that in the ITT population, PFS utility values, discount rate, capecitabine cost, PD utility values, and pembrolizumab cost showed significant associations with outcomes, while adverse event disutility values and second-line treatment costs exhibited moderate or low correlations. However, none of these variables yielded an ICER below the WTP threshold.

One-way and probabilistic sensitivity analysis results are presented in Figure 2. The cost-effectiveness acceptability curve at a WTP threshold of $40,334.05/QALY indicated a 0% probability that pembrolizumab plus trastuzumab and chemotherapy as first-line treatment for HER2-positive GC or GEJC adenocarcinoma would be cost-effective. This conclusion was further supported by scatter plot results.

**Figure 2:**
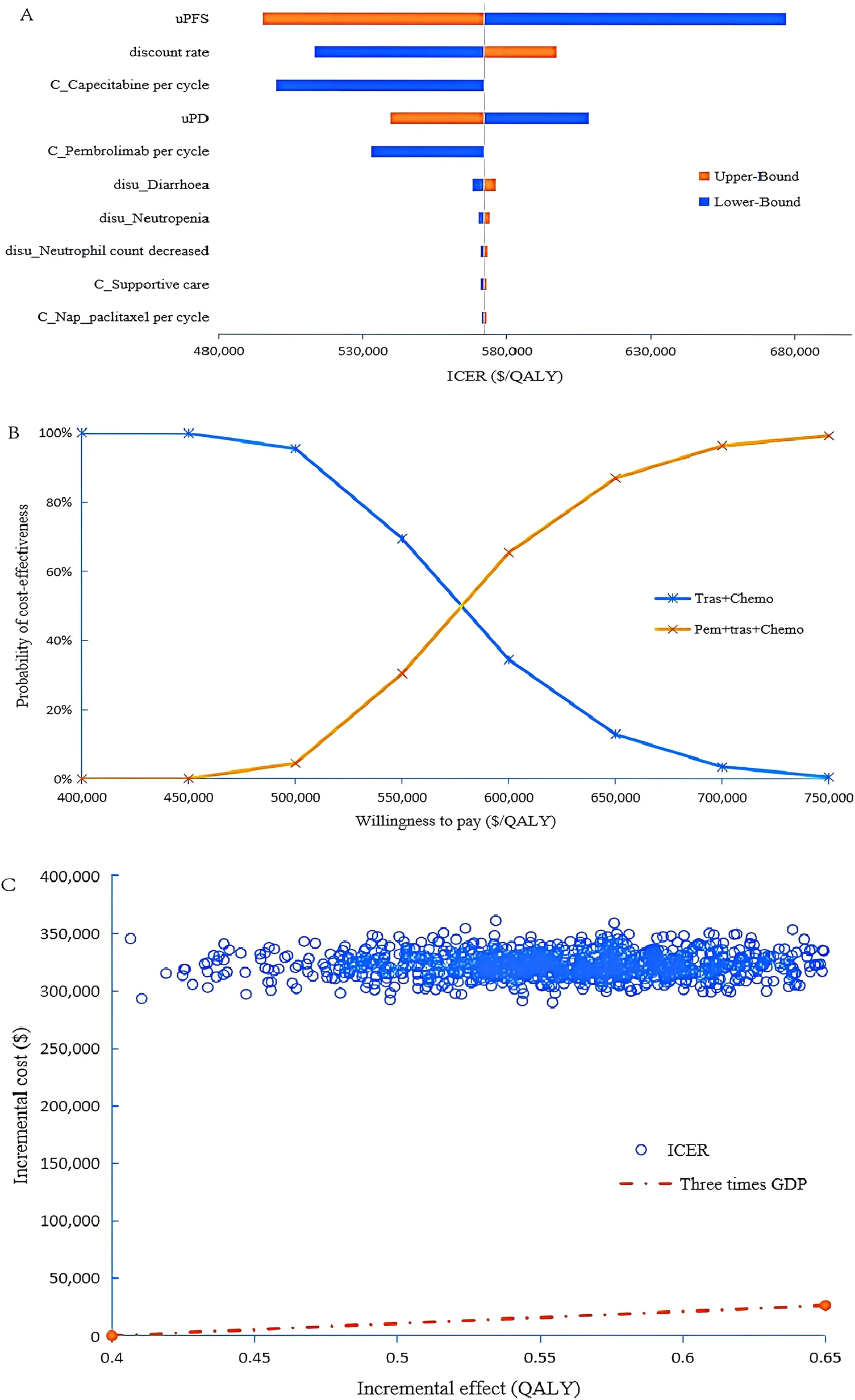
Results of one-way, probabilistic sensitivity analyses of the ITT population.

The one-way sensitivity analysis for the PD-L1 ≥ 1 subgroup revealed that PFS utility values, discount rate, per-cycle capecitabine cost, PD utility values, and pembrolizumab price exerted significant influence on outcomes, while adverse event disutility and second-line treatment costs demonstrated moderate or minimal impact. Nevertheless, the overall results remained robust. These findings are illustrated in Figure 3.

**Figure 3:**
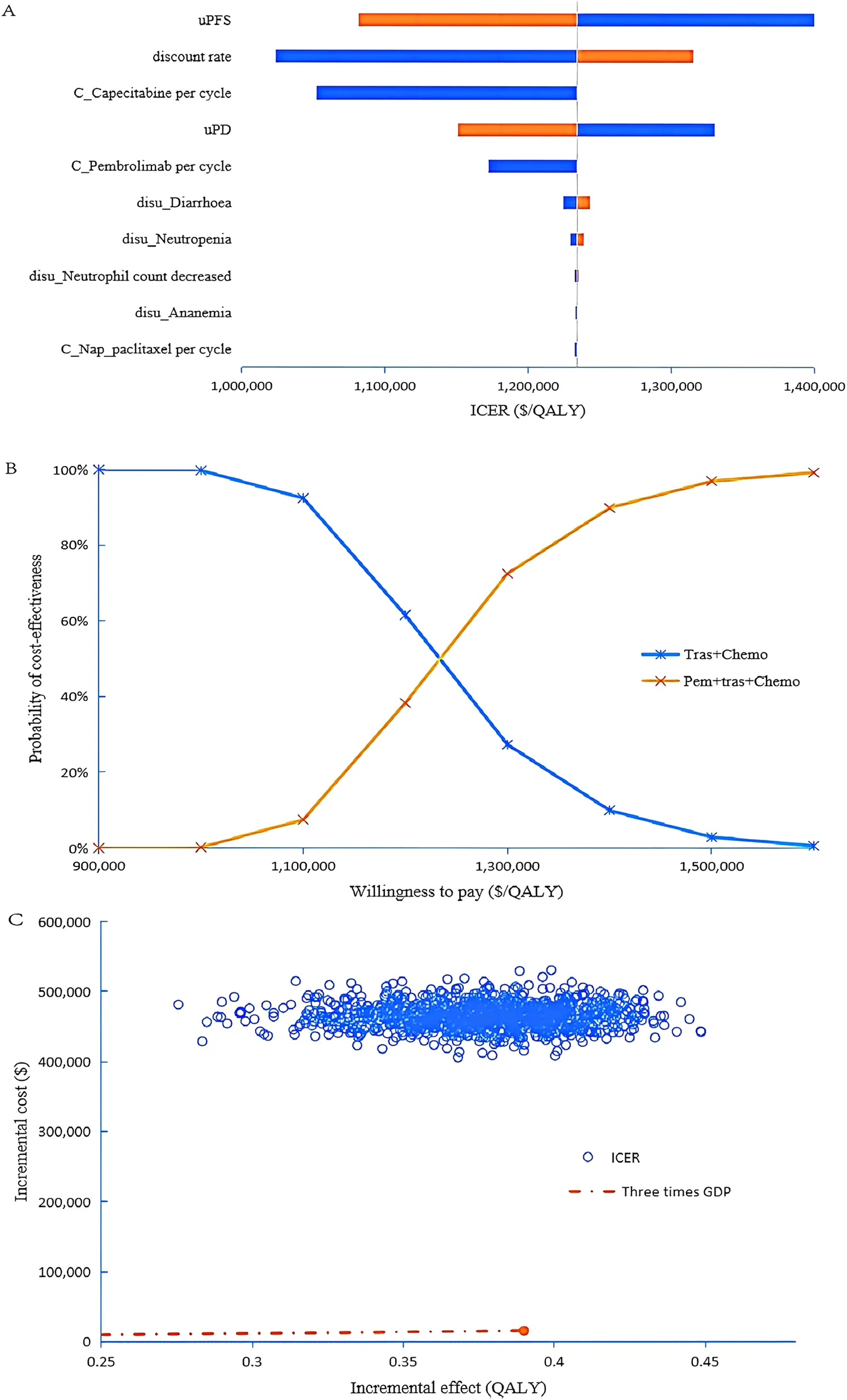
Results of one-way, probabilistic sensitivity analyses of the PD-L1≥1 population.

Probabilistic sensitivity analysis results demonstrated that at the WTP threshold of $40,334.05 per QALY, the probability of pembrolizumab plus trastuzumab and chemotherapy being cost-effective as first-line treatment for HER2-positive GC/GEJC adenocarcinoma was 0%. This conclusion was further corroborated by scatter plot analysis.

## Discussion

Pembrolizumab plus trastuzumab combined with chemotherapy has garnered significant attention for treating HER2-positive GC or GEJC adenocarcinoma. A recent meta-analysis (24) demonstrated that pembrolizumab significantly improves overall survival (OS) and objective response rates compared to chemotherapy alone in gastric and gastroesophageal junction cancers, while maintaining favorable tolerability. These clinical benefits suggest pembrolizumab's potential utility for HER2-positive GC/GEJC adenocarcinoma patients. However, the high cost of pembrolizumab presents substantial challenges for healthcare sustainability, particularly given the global burden of over 1.23 million new gastric cancer cases in 2021 (25). As emphasized in previous studies (26, 27), comprehensive cost-effectiveness evaluations of pembrolizumab for HER2-positive GC/GEJC adenocarcinoma are crucial for informing policy decisions and optimizing healthcare resource allocation. Consequently, a thorough pharmacoeconomic assessment of pembrolizumab in this indication is warranted.

The findings of our study revealed that the ICERs for pembrolizumab or placebo combined with trastuzumab and chemotherapy in the ITT population and PD-L1 ≥ 1 population with GC or GEJC adenocarcinoma were $571,997.42 and $1,178,714.74, respectively, both exceeding China's WTP threshold of $40,334.05 per QALY. In the one-way sensitivity analysis, PFS utility values, discount rates, capecitabine cycle costs, PD utility values, and pembrolizumab pricing significantly impacted the base-case results for both populations. However, none of these factors reduced the ICER below the WTP threshold, demonstrating the robustness of our model. Probabilistic sensitivity analysis indicated that when using three times China's per capita GDP as the WTP threshold, the probability of pembrolizumab combined with trastuzumab and chemotherapy being cost-effective as first-line therapy for HER2-positive GC or GEJC adenocarcinoma patients was 0%, this outcome aligns with the results reported in earlier literature (28, 29). This lack of economic advantage may stem from the high cycle costs of pembrolizumab, trastuzumab, and capecitabine, combined with our lifetime simulation of medical expenditures, which likely contributed to the unfavorable cost-effectiveness profile of this regimen.

A recent study by Lang et al. (30), based on the KEYNOTE-859 trial, assessed the cost-effectiveness of pembrolizumab combined with chemotherapy versus chemotherapy alone for HER-2-negative advanced GC or GEJC from the Chinese healthcare system perspective. Their results were consistent with ours, demonstrating that pembrolizumab plus chemotherapy was not cost-effective compared to chemotherapy alone, with an ICER of $76,936.60/QALY. In the PD-L1 ≥ 1 or PD-L1 ≥ 10 subgroups, the ICERs were $72,762.68/QALY and $34813.70/QALY, respectively. Zhu et al. (31) conducted a cost-effectiveness analysis comparing pembrolizumab and nivolumab, each combined with chemotherapy, for gastroesophageal junction tumors from the perspectives of the Chinese, U.S., and U.K. healthcare systems. Their findings suggested that, from the Chinese payer's perspective, pembrolizumab plus chemotherapy yielded more QALYs at a lower cost than nivolumab plus chemotherapy. This result differs from our study, possibly due to several factors: (1) Zhu et al. compared pembrolizumab with nivolumab, and although the chemotherapy regimens were the same, differences in patient populations and tumor status may have introduced uncertainty in the modeling process; (2) our study evaluated the cost-effectiveness of PD-1 + HER-2 dual blockade for HER-2-positive GC or GEJC adenocarcinoma, which included additional trastuzumab costs; and (3) the time horizons of the two studies differed. These factors may explain the discrepancies in conclusions. Uncertainty analyses confirmed the robustness of our results.

One strength of this study is that the survival efficacy inputs including overall survival and progression-free survival curves were derived from KEYNOTE-811, a global phase III trial recruiting participants at 168 medical centers covering 20 countries. The multinational study population ensures that the fitted parametric survival functions shown in Supplementary Table S3 can reflect the natural disease trajectory and therapeutic efficacy among worldwide patients with HER2-positive gastric or gastroesophageal junction adenocarcinoma, eliminating extrapolation bias caused by small-sample efficacy data limited to a single country. Additionally, our study employed a partitioned survival model to directly derive the proportions of patients in different health states from the OS and PFS curves, minimizing reliance on assumptions. Furthermore, we conducted a subgroup analysis specifically for patients with PD-L1 ≥ 1, providing valuable evidence on treatment benefits based on PD-L1 expression levels.

This study has several limitations. First, it only compared pembrolizumab or placebo plus trastuzumab and chemotherapy without evaluating the cost-effectiveness of other immune checkpoint inhibitors with proven benefits in GC or GEJC adenocarcinoma, such as nivolumab, sintilimab, and toripalimab. Due to the lack of head-to-head clinical trials among these immunotherapies, future studies could conduct indirect economic evaluations using network meta-analysis if sufficient data become available. Second, the study adopted a lifetime simulation horizon, with survival extrapolation beyond the follow-up period based on parametric survival models. Consequently, the accuracy of the findings may be influenced by the extrapolation method. Real-world long-term follow-up data are needed to validate these results. Third, since KEYNOTE-811 did not report utility values specifically for HER2-positive GC or GEJC adenocarcinoma patients, the utility values in this study were derived from previously published research, which may introduce potential bias. Fourth, to simplify the model, only grade ≥3 treatment-related adverse events (AEs) were considered, while grade 1–2 AEs and long-term side effects were excluded. This approach might lead to an underestimation of costs and disutility. Nevertheless, uncertainty analyses confirmed the robustness of the model results, demonstrating that the findings are generally reliable and robust.

Although clinical trial data demonstrated that pembrolizumab plus trastuzumab and chemotherapy significantly improved OS and PFS as first-line treatment for HER2-positive GC or GEJC adenocarcinoma, our study indicates that this regimen lacks cost-effectiveness advantages in both the ITT population and PD-L1 ≥ 1 subgroup within the context of Chinese healthcare system. Future validation of these model results may be achieved through long-term follow-up data from real-world studies.

## Conclusions

From the perspective of Chinese healthcare system, the pembrolizumab plus trastuzumab and chemotherapy regimen did not demonstrate cost-effectiveness advantages compared with placebo plus trastuzumab and chemotherapy in HER2-positive GC or GEJC adenocarcinoma patients, regardless of whether in the ITT population or PD-L1 subgroups. These findings provide important evidence for clinical decision-making.

## Author's note

Patients and/or the public were not involved in the design, or conduct, or reporting, or dissemination plans of this research.

## Data Availability

The original contributions presented in the study are included in the article. Further inquiries can be directed to the corresponding authors.
