# Supplementary material for: Cost-effectiveness analysis of pembrolizumab plus trastuzumab and chemotherapy as first-line treatment for HER2-positive gastric or gastroesophageal junction adenocarcinoma
Source: Front Med (Lausanne). 2026 Sep 15;13:1905243. doi: 10.3389/fmed.2026.1905243 (PMC13619512; doi:10.3389/fmed.2026.1905243)
Supplement: Supplementary file 1 [file Table1.docx]

Supplementary Material

# 1 Supplementary Figures

**Supplementary Figure 1.**  Raw K-M curves and best-fit curves for OS (A) and PFS (B) in the pembrolizumab/placebo group in the ITT population

**Supplementary Figure2** Raw K-M curves and best-fit curves for OS (A) and PFS (B) in the pembrolizumab/placebo group in the PD-L1≥1 population

# 2 Supplementary Figures

- **Supplementary Table 1**. Baseline characteristics between the Pembrolizumab and Chemotherapy groups

|  | Pembrolizumab（n=350） | Chemotherapy  （n=348） |
| --- | --- | --- |
| **Age, years** | 62.0 (54.0–69.0) | 63.0 (55.0–70.0) |
| **Age group** |  |  |
| ≥65 years | 145（41%） | 156（45%） |
| **Sex** |  |  |
| Male | 284（81%） | 280（80%） |
| Female | 66（19%） | 68（20%） |
| **Race** |  |  |
| White | 218（62%） | 212（61%） |
| Asian | 119（34%） | 121（35%） |
| American Indian or Alaska Native | 5（1%） | 6（2%） |
| Black or African American | 2（1%） | 2（1%） |
| Multiple | 6（2%） | 5（1%） |
| **ECOG performance status** |  |  |
| 0 | 146（42%） | 146（42%） |
| 1 | 204（58%） | 202（58%） |
| **Primary location at diagnosis** |  |  |
| Gastro-oesophageal junction | 110（31%） | 122（35%） |
| Stomach | 240（69%） | 226（65%） |
| **PD-L1 status** |  |  |
| CPS ≥1 | 298 (85%) | 296 (85%) |
| CPS <1 | 52 (15%) | 52 (15%) |
| **HER2 status, n (%)** |  |  |
| IHC 1+ | 1 (<1%) | 1 (<1%) |
| IHC 2+ ISH equivoca | 0 | 1 (<1%) |
| IHC 2+ ISH negative* | 1 (<1%) | 1 (<1%) |
| IHC 2+ ISH positive | 62(18%) | 84(24%) |
| IHC 3+ | 286 (82%) | 261 (75%) |
| **Treatment-related adverse events（Grade ≥3）** |  |  |
| Diarrhoea | 31 (9%) | 27 (8%) |
| Anemia | 21 (6%) | 20 (6%) |
| Neutrophil count decreased | 28 (8%) | 30 (9%) |
| Platelet count decreased | 22 (6%) | 23 (7%) |
| Neutropenia | 22 (6%) | 16(5%) |

Abbreviations: *Some patients with IHC 1+ and IHC 2+ ISH-negative tumours were included per investigator assessment; ECOG=Eastern Cooperative Oncology Group; IHC=immunohistochemistry test score; ISH=in-situ hybridisation

- **Supplementary Table 2**. Summary of statistical goodness-of-fit of KM curves in KEYNOTE-811 trial

|  | Exponential | Gamma | Gompertz | Weibull | Log-logistic | Log-normal |
| --- | --- | --- | --- | --- | --- | --- |
| **ITT Population** |  |  |  |  |  |  |
| OS curve of the pembrolizumab group |  |  |  |  |  |  |
| AIC | 2609.882 | 2605.128 | 2605.624 | 2609.827 | 2568.053 | **2567.658** |
| BIC | 2613.740 | 2612.844 | 2613.340 | 2617.543 | 2575.768 | **2575.374** |
| PFS curve of the pembrolizumab group |  |  |  |  |  |  |
| AIC | 2274.751 | 2276.271 | 2245.404 | 2276.021 | **2214.596** | 2215.376 |
| BIC | 2278.609 | 2283.987 | 2253.120 | 2283.736 | **2222.311** | 2223.092 |
| OS curve of the placebo group |  |  |  |  |  |  |
| AIC | 2720.563 | 2718.153 | 2718.448 | 2721.284 | **2689.574** | 2698.106 |
| BIC | 2724.415 | 2725.857 | 2726.153 | 2728.988 | **2697.278** | 2705.810 |
| PFS curve of the placebo group |  |  |  |  |  |  |
| AIC | 2138.798 | 2135.758 | 2129.384 | 2140.221 | **2084.138** | 2087.998 |
| BIC | 2142.650 | 2143.463 | 2137.089 | 2147.925 | **2091.842** | 2095.703 |
| **PD-L1≥1 Population** |  |  |  |  |  |  |
| OS curve of the pembrolizumab group |  |  |  |  |  |  |
| AIC | 2028.661 | 2019.849 | 2030.455 | 2024.422 | **2001.506** | 2003.498 |
| BIC | 2032.358 | 2027.243 | 2037.849 | 2031.816 | **2008.900** | 2010.893 |
| PFS curve of the pembrolizumab group |  |  |  |  |  |  |
| AIC | 1942.324 | 1944.297 | 1912.805 | 1942.769 | **1891.251** | 1891.568 |
| BIC | 1946.022 | 1951.692 | 1920.199 | 1950.163 | **1898.645** | 1898.962 |
| OS curve of the placebo group |  |  |  |  |  |  |
| AIC | 2192.233 | 2190.583 | 2192.088 | 2192.965 | **2171.101** | 2177.368 |
| BIC | 2195.924 | 2197.963 | 2199.469 | 2200.345 | **2178.481** | 2184.748 |
| PFS curve of the placebo group |  |  |  |  |  |  |
| AIC | 1868.624 | 1868.370 | 1855.501 | 1870.624 | **1818.294** | 1823.109 |
| BIC | 1872.315 | 1875.750 | 1862.882 | 1878.004 | **1825.675** | 1830.490 |

Abbreviations: AIC, Akaike information criterion; BIC, Bayesian information criterion; OS, overall survival; K-M: Kaplan-Meier; PFS, progression-free survival; ITT, intention-to-treat

- **Supplementary Table 3.** Survival parameters for the two treatment strategies

| **regimens** | **K-M curve** | **model** | **Survival parameters** |
| --- | --- | --- | --- |
| **ITT Population** |  |  |  |
| Pembrolizumab+trastuzumab+chemotherapy group | OS | Log-normal | Meanlog=3.421 Sdlog=1.097 |
| Pembrolizumab+trastuzumab+chemotherapy group | PFS | Log-logistic | Scale=0.015 Shape=1.507 |
| trastuzumab+chemotherapy group | OS | Log-logistic | Scale=0.007 Shape=1.565 |
| trastuzumab+chemotherapy group | PFS | Log-logistic | Scale=0.015 Shape=1.681 |
| **PD-L1≥1 Population** |  |  |  |
| Pembrolizumab+trastuzumab+chemotherapy group | OS | Log-logistic | Scale=0.004 Shape=1.615 |
| Pembrolizumab+trastuzumab+chemotherapy group | PFS | Log-logistic | Scale=0.017 Shape=1.458 |
| trastuzumab+chemotherapy group | OS | Log-logistic | Scale=0.008 Shape=1.535 |
| trastuzumab+chemotherapy group | PFS | Log-logistic | Scale=0.017 Shape=1.667 |

Abbreviations:OS, overall survival; K-M: Kaplan-Meier; PFS, progression-free survival; ITT, intention-to-treat
